# On the Identity or Non-identity of Typhoid Fever

**Published:** 1850-06

**Authors:** W. Jenner

**Affiliations:** Professor of Pathological Anatomy, University College, London


*On the Identity or Non-identity of Typhoid Fever, Typhus Fever, and Relapsing Fever.* By W. Jenner, M. D., Professor of Pathological Anatomy, University College, London, (*Proceedings of the Royal Medical and Chirurgical Society*, Dec. 11, 1849.)—The author, at the commencement of his paper, remarks that, for many years, small-pox, measles, and scarlet fever were confounded under one name, and that it was only after the publication of Dr. Withering's essay, that measles and scarlet fever were regarded as distinct affections—i. e., distinct as to their course, their symptoms, their lesions, and their causes. Typhus fever, typhoid fever, and relapsing fever are yet by many looked on but as varieties of one disease. But the writings of Dr. Gerhard, M. Valleix, and Dr. A. P. Stewart, have rendered it highly probable that typhoid fever and typhus fever are absolutely distinct from each other—two species of disease, and not varieties of one affection. In the Monthly Journal of Medicine of the present year, the author has analyzed the course, symptoms, and lesions of structure found after death in a certain number of cases of fever, and this analysis, he thinks, proves that, as regards their course, symptoms, and lesions, no two diseases can be more distinct than typhus and typhoid fever. But small pox, measles, and scarlet fever differ also in respect of their exciting cause, which, in the case of each of these diseases, is specific. In like manner, typhoid fever, typhus fever, and relapsing fever must require for their production the application of distinct specific causes, if they be distinct diseases. To inquire whether the specific cause of each of these diseases is distinct, or whether the cause of all these is the same, is the author's object in the present paper. He first describes the peculiarities of the course and symptoms of relapsing fever, and of the skin eruption of typoid fever and of that of typhus fever, on which the diagnosis of these diseases rests. He then gives three tables, showing all the instances in which two or more cases of fever were admitted from one house into the London Fever Hospital in the years 1847, 1848, and 1849; the age, sex, and degree of intimacy of the individuals, as well the nature of the disease under which they labored; and for the years 1848 and 1849, the number of all cases of fever admitted into the Fever Hospital during the separate months, with the rash of typhoid fever and that of typhus fever respectively. The results exhibited in these tables are: 1st. That, in 1847, there were five instances of the admission of two or more cases of typhus fever from the same house, two instances of the admission of two cases of typhoid fever from the same house, and five instances of the admission of two cases of relapsing fever from the same house, and not a single instance of cases of the three diseases, or even of two of them, coming from the same locality. 2d. That, in 1848, two or more cases of typhus fever were admitted from each of thirty-three houses, and two cases of typhoid fever from each of four houses; while there was only one instance of a case of typhus and one of typhoid fever being admitted from one house; and in this exceptional instance there is reason to believe the patients received their diseases from different sources. 3d. That, in 1849, two or more cases of typhus fever were admitted from each of eighteen separate localities; and two or more cases of typhoid fever from each of four localities; while in not a single instance was a case of typhoid fever and a case of typhus fever admitted from the same house. 4th. That, in 1847, the relapsing fever, typhoid fever, and typhus fever, and, in 1848 and 1849, the typhus and typhoid fevers, prevailed simultaneously in the metropolis; and, nevertheless, that the cases of these several diseases came to the hospital from distinct localities. In 1848, there were received into the fever hospital 118 cases of fever with the rash of typhoid fever, and 390 with the rash of typhus fever; in 1849, 118 with the rash of typhoid fever, and 143 with the rash of typhus fever. As, therefore, about one-fourth of all the cases of fever admitted in 1848, and nearly half of those admitted in 1849, were cases of typhoid fever, the author argues that, in the numerous instances in which two or more cases were admitted from one locality, cases of typhoid fever ought to have been mingled indifferently with cases of typhus fever, in about their proportion in the two years, if the cause of the two diseases were identical; while, as has been shown, from all the localities which yielded cases of typhus, there came but one case of typhoid fever. He remarks, moreover, that an increase of the epidemic prevalence of one of these kinds of fevers had no influence in increasing or diminishing the absolute number of cases of the other kind of fever; that no transition-cases were observed, marking the passage of one epidemic constitution into another; that the rash of typhoid fever and that of typhus fever were not modified in their characters by variations in the prevalence of other kinds of fever; and that the absence or presence of lesion of Peyer's patches and the mesenteric glands always corresponded with the symptoms of the particular cases during life, and did not depend on the epidemic constitution. The author then adduces some particular instances, in which a succession of cases, coming from the same locality, or apparently arising from the same cause, all presented the same characters. And, in conclusion, he remarks that the facts contained in this paper appear to him to prove, incontestably, that the specific causes of typhus and typhoid fevers are absolutely different from each other; and to render it in the highest degree probable that the specific cause of relapsing fever is different from that of either of the two former.

In the debate which followed, Dr. Jenner stated that, like others, he had seen cases of typhoid fever in which there was no eruption; and he spoke now of the spots, when present, only as a means of diagnosis. It must be remembered that in scarlet fever and measles the exanthem was occasionally absent; and this would serve to point out the inconsistency of those who sought to associate typhus and typhoid fevers, because occasionally there was no eruption. The mulberry spots in typhus, at first, in many cases, bore a close resemblance to the rose-colored spots of typhoid fever; so that in a few cases they might be regarded as exactly similar. His experience, however, fortified by 2,000 cases, enabled him to assert that if, by the eighth day of the disease, the eruption presented the true typhous character, then, if death ensued, intestinal lesions would not be discovered. Dr. Baly's cases, to be of any service in determining the diagnostic value of the eruption, must be confined to the fatal cases, where the after-death appearances were described: and these appearances had not been mentioned by him. With regard to the opinions of Dr. Crawford, respecting the influence of impurity of the atmosphere and locality on the eruption, he might state that the Fever Hospital received patients from all parts of London, and its neighborhood. Personal observation of houses, in these various localities, had not enabled him to observe any difference in their hygienic conditions. It must be self-evident, that no very great impurity of atmosphere existed in the Fever Hospital, when no such change as that mentioned by Dr. Crawford, he (Dr. Jenner) could distinctly deny, ever occurred. He agreed with Dr. West, that infantile remittent fever was really typhoid, and never gave rise to typhus. He reminded Dr. Stewart that, in his published essays, he had described a fatal case of typhoid fever from relapse, in which the eruption appeared for the second time. The fact of Dr. Christison having suffered three times from typhus fever was no evidence that typhus fever occurred more frequently than typhoid fever in the same person; because he might have had the three different forms of fever under discussion, all of which Dr. Christison might regard as simple continued fever. After some remarks on the concurring testimony of Dr. Gerhard, Dr. Shattuck, Dr, Stewart, and himself, respecting these fevers, he observed that it was a frequent occurrence, in the Fever Hospital, for patients affected with typhus and typhoid fevers to lie side by side in the same ward, each disease preserving its own peculiar symptoms.

